# A giant periosteal chondroma of the distal femur successfully reconstructed with synthetic bone grafts and a bioresorbable plate: a case report

**DOI:** 10.1186/1477-7819-12-354

**Published:** 2014-11-22

**Authors:** Yoshinori Imura, Atsuo Shigi, Hidetatsu Outani, Kenichiro Hamada, Hiromi Tamura, Eiichi Morii, Akira Myoui, Hideki Yoshikawa, Norifumi Naka

**Affiliations:** Department of Orthopaedic Surgery, Osaka University Graduate School of Medicine, 2-2 Yamadaoka, Suita, Osaka, 565-0871 Japan; Department of Pathology, Osaka University Graduate School of Medicine, 2-2 Yamadaoka, Suita, Osaka, 565-0871 Japan

**Keywords:** bioresorbable plate, distal femur, intensive curettage, intralesional resection, periosteal chondroma, synthetic bone grafts

## Abstract

Periosteal chondromas are rare benign cartilaginous tumors that arise adjacent to the cortex beneath the periosteum. These lesions are usually slow-growing and rarely exceed 3 cm in the greatest dimension. Here, we describe a 17-year-old boy who had a giant periosteal chondroma of the right distal femur, which was treated with intralesional resection and intensive curettage. In addition, we report a novel application of a bioresorbable plate in the management of the large bone defect after resection of a benign bone tumor.

## Background

Periosteal chondromas (juxtacortical chondromas) were first described by Lichtenstein and Hall in 1952 [1]. They are relatively uncommon, benign, hyaline cartilage tumors that account for less than 2% of chondromas and are generally seen in men in their second and third decades of life [2–6]. These lesions develop adjacent to the cortical surface of bone beneath the periosteal membrane and often arise at the metaphyses of long tubular bones, most commonly the humerus followed by the femur and tibia [3–6]. They are slow-growing tumors and usually smaller than 3 cm [7, 8].

Here, we report a case of a giant periosteal chondroma involving the right distal femur in a 17-year-old boy. We treated this patient by intralesional resection and intensive curettage, resulting in a large bone defect. We successfully performed reconstruction of the defect using interconnected porous calcium hydroxyapatite (IP-CHA) and a bioresorbable plate.

## Case presentation

A 17-year-old boy presented to our hospital with mild pain in his right thigh, which he had experienced for the previous month. The patient stated that 6 years earlier he had hit this same thigh and felt pain and swelling at that time. Then he visited the local doctor and a mass on the anterior surface of his right distal femur was revealed by plain radiographs. The symptoms gradually decreased with time and he had received a follow-up examination from the doctor. The patient had an otherwise unremarkable past medical history.

**Figure 1:**
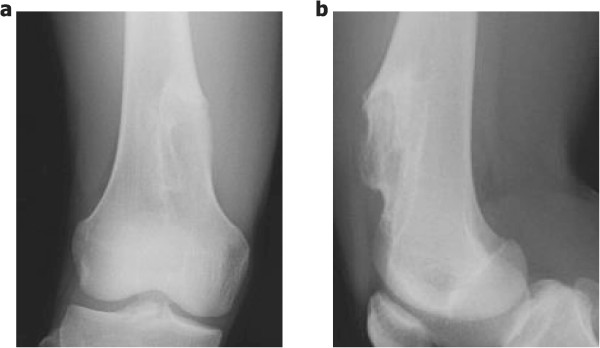
**Preoperative plain radiographs. (a)** Anteroposterior and **(b)** lateral radiographs showing cortical erosion, saucerization of the underlying cortex, and a rim of sclerosis on the anterior surface of the right distal femur.

**Figure 2:**
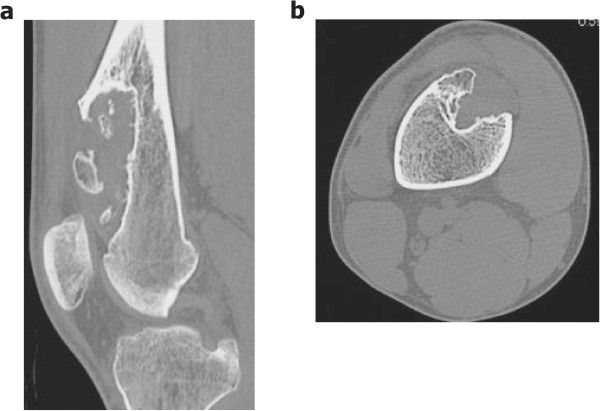
**Preoperative CT. (a)** Sagittal and **(b)** axial scans, showing an extraskeletal mass with focal calcification on the anterior surface of the right distal femur.

**Figure 3:**
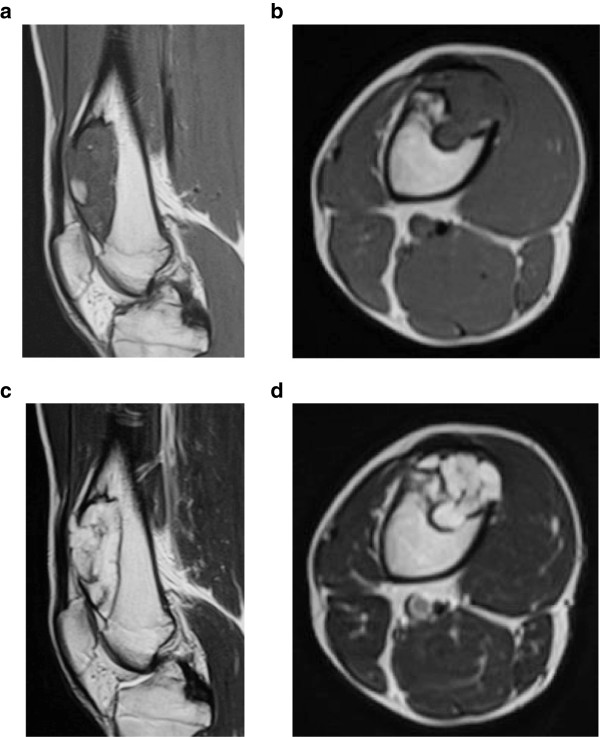
**Preoperative MRI. (a)** Sagittal and **(b)** axial T1-weighted images, showing a low-intensity mass on the anterior surface of the right distal femur. **(c)** Sagittal and **(d)** axial T2-weighted images, showing a high-intensity mass. No evidence of marrow invasion was seen.

At the first presentation to our clinic, physical examination revealed a firm, nontender mass with ill-defined margins at the anterior aspect of his right thigh. The mass was neither freely moveable nor adherent to the skin. Range of motion was decreased at the right knee joint. Plain radiographs showed that the lesion was located on the anterior surface of the right distal femur with cortical erosion, saucerization of the underlying cortex, and a rim of sclerosis (Figure 1a,b). In addition, computed tomography (CT) showed an extraskeletal mass with focal calcification (Figure 2a,b). Magnetic resonance imaging (MRI) showed low intensity in the T1-weighted image and high intensity in the T2-weighted image (Figure 3a-d). The mass measured 8.9 cm long by 2.7 cm wide with a maximum height of 3.1 cm. The mass did not appear to invade the intramedullary canal. A preoperative diagnosis of periosteal chondroma or low-grade (grade 1) periosteal chondrosarcoma was made. As both of these tumors are frequently less aggressive than high-grade (grade 2 or 3) chondrosarcomas, we elected to perform intralesional resection and intensive curettage after intraoperative pathological consultation instead of biopsy.

**Figure 4:**
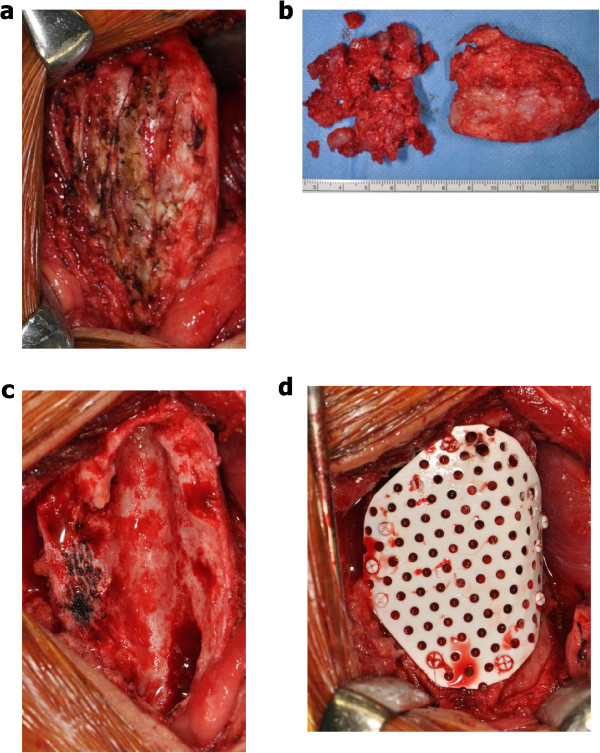
**Intraoperative findings. (a)** The tumor located on the anterior aspect of the right distal femur. **(b)** Macroscopic examination of the specimen treated by intralesional resection and intensive curettage. **(c)** The large bone defect after resection of the tumor. **(d)** Appearance of the lesion after reconstruction of the defect with IP-CHA and a bioresorbable plate.

**Figure 5:**
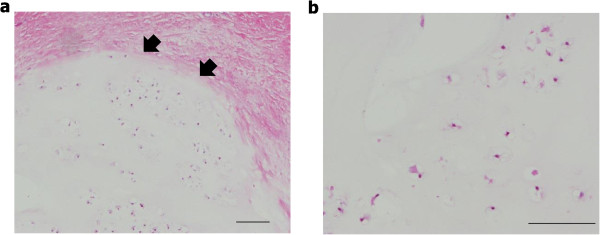
**Histology of the resected specimen (H & E stain). (a)** Benign hyaline cartilage tumor covered with periosteum. Arrow heads indicate the border between the lesion and the periosteum. **(b)** Hypocellular cartilage tumor without cytologic atypia and mitosis. Scale bars: 100 μm.

We operated according to the preoperative surgical plan. The tumor was well-circumscribed without invasion into the muscle or subcutaneous tissue (Figure 4a). Macroscopically, the periosteal tumor consisted of an oval, well-circumscribed, cartilaginous tumor, measuring 9 × 3 × 3 cm in greater dimension (Figure 4b). An intraoperative rapid pathological diagnosis of periosteal chondroma or low-grade periosteal chondrosarcoma was made. After resection and intensive curettage of the entire lesion, we noted a large bone defect of the anterior aspect of the distal femur (Figure 4c). We then grafted IP-CHA (Neobone®; MMT Co. Ltd., Osaka, Japan) [9] to the defect and covered it with a bioresorbable plate (Super FIXSORB® MX Mesh; Takiron Co. Ltd., Osaka, Japan) (Figure 4d). Histological examination of the resected specimen showed a hyaline cartilage tumor arranged in a lobular pattern and covered by periosteum without cytologic atypia or mitosis (Figure 5a,b). The margins of the mass were circumscribed, with no evidence of invasion or permeation of adjacent structures. The final pathological diagnosis was periosteal chondroma.

**Figure 6:**
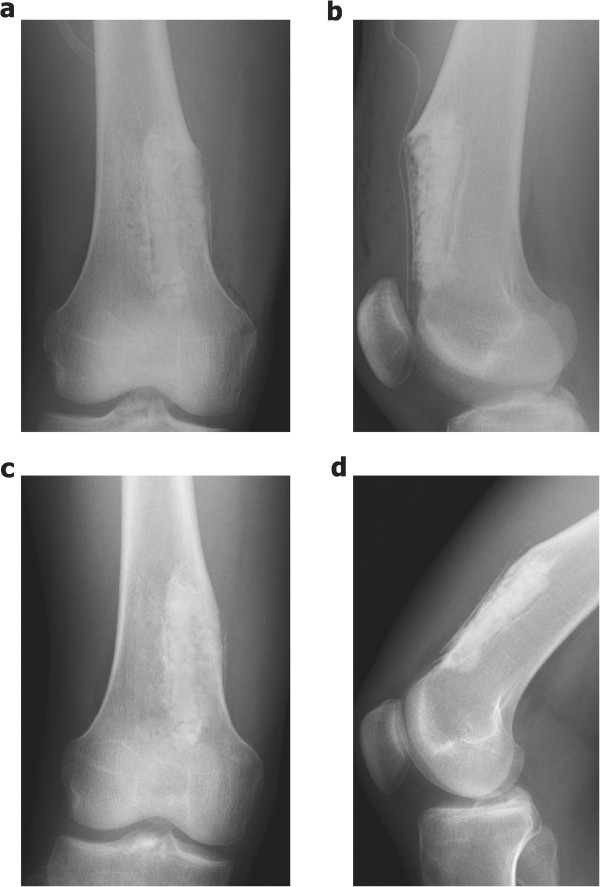
**Postoperative plain radiographs. (a)** Anteroposterior and **(b)** lateral postoperative routine radiographs showing the bone defect filled with IP-CHA and covered with a bioresorbable plate. **(c)** Anteroposterior and **(d)** lateral radiographs at 12-month follow-up showing no local recurrence of the tumor. Particles of IP-CHA were almost consolidated.

The patient’s postoperative course was uneventful and he was discharged on postoperative day 14. Six months after surgery, the range of motion in his right knee was fully recovered and he returned to sports, including football and baseball. Final radiographs revealed that particles of IP-CHA were almost consolidated after the operation (Figure 6a-d). There has been no sign of local recurrence during the 12 months since the surgical resection.

## Discussion

Benign and malignant periosteal chondroid tumors are now recognized as distinct disease entities. The differential diagnosis to distinguish between periosteal chondromas and periosteal chondrosarcomas is necessary for avoiding aggressive and inappropriate treatment. There are three fundamental radiographic features of periosteal chondromas, including scalloping or remodeling of the adjacent bony cortex, the presence of cartilaginous matrix that appears as small dots and ringlets of calcification, and a possible soft tissue mass component [8, 10, 11]. Although scalloping and sclerosis of the cortex can be similar in the two lesions, periosteal chondrosarcomas tend to invade the underlying bone, form no reactive bone underlying the cortical lesion, and present a large mass in the soft tissue [12]. In this case, imaging features of the tumor were characteristic of a periosteal chondroma but not of a periosteal chondrosarcoma.

It has been reported that the median size of periosteal chondrosarcomas was considerably larger than that of chondromas [7, 8]. In this case, the size of the tumor was approximately 9 cm in the longest diameter but had taking more than 6 years to grow to this size, suggesting that the tumor might be a giant periosteal chondroma rather than a slow-growing low-grade periosteal chondrosarcoma. In fact, the final pathological diagnosis was periosteal chondroma.

Nevertheless, it is frequently difficult to differentiate periosteal chondromas from low-grade periosteal chondrosarcomas. Wide excision is the treatment of choice in high-grade chondrosarcomas but may result in increased morbidity and functional deficit [13, 14]. As the potential for local recurrences and metastases in low-grade periosteal chondrosarcomas is extremely low, the necessity of a wide margin is controversial for them [13–20]. Many authors described that less extensive surgery was indicated for low-grade chondrosarcomas, resulting in a good functional outcome [15, 19–21]. Conversely, the recommended treatment for periosteal chondroma is intralesional or marginal resection [4, 6]. According to a literature review of Lewis *et al*. [4], out of 165 cases of periosteal chondromas, only six cases of local recurrence were reported, a recurrence rate of 3.6%. Mora *et al*. [22] demonstrated that the reported cases of local recurrence could be attributed to inadequate intralesional excision. No malignant transformation, metastasis, or multiple lesions have been reported. In the present case, intraoperative rapid pathological diagnosis of periosteal chondroma or low-grade periosteal chondrosarcoma was made and thus we carried out intralesional resection and intensive curettage to the entire lesion.

A large bone defect occurring after resection of the tumor is frequently problematic. Recently, resorbable bone devices have been developed [23, 24]. Super FIXSORB MX® is a bioactive and totally resorbable osteosynthetic bone fixation device [24]. This device is made from composites of unsintered hydroxyapatite particles and poly L-lactide, which has been reinforced using a unique compression forging process [24]. This device has a modulus of elasticity close to that of natural cortical bone and retains high strength during the period required for bone healing [24–26]. It also shows optimal degeneration, resorption behavior, and osteoconductivity, thus obviating the need for implant removal [24–27]. In addition, in this case, bone grafting alone was not sufficient to reconstruct the bone defect and so we used a bioresorbable plate to prevent particles of IP-CHA from leaking out of the huge defect. The range of motion in the knee was fully recovered, suggesting that the plate did not cause severe adherence or tightness of the quadriceps femoris.

Bioresorbable bone devices have many potential applications in various clinical fields, such as craniofacial, trauma, and spine surgery [27–29]. It has been reported that seven patients with benign bony tumors of the anterior cranial vault and orbit underwent simultaneous bony excision and reconstruction with alloplastic hard tissue replacement implants [30]. However, to the best of our knowledge, this is the first report of reconstruction of the large bone defect after removal of the periosteal chondroma arising in the long tubular bone using synthetic bone grafts and a bioresorbable plate. This novel reconstruction method should be effective for large bone defects after resection and curettage of common benign bone tumors.

## Conclusions

We present a case of a giant periosteal chondroma in the right distal femur of a 17-year-old boy. We successfully treated the patient by intralesional resection and intensive curettage of the tumor and performed reconstruction of the large bone defect by grafting synthetic bone grafts and using a bioresorbable plate.

## Consent

Written informed consent was obtained from the patient for publication of this case report and any accompanying images. A copy of the written consent is available for review by the editor-in-chief of this journal.

## Abbreviations

CT: computed tomography
H & E: hematoxylin and eosin
IP-CHA: interconnected porous calcium hydroxyapatite
MRI: magnetic resonance imaging.
